# Teachers’ Psychological Needs Satisfaction and Thwarting: Can They Explain Students’ Behavioural Engagement in Physical Education? A Multi-Level Analysis

**DOI:** 10.3390/ijerph17228573

**Published:** 2020-11-19

**Authors:** Javier Coterón, Evelia Franco, Carmen Ocete, Javier Pérez-Tejero

**Affiliations:** 1 Departamento de Ciencias Sociales de la Actividad Física, del Deporte y del Ocio, Facultad de Ciencias de la Actividad Física y del Deporte—INEF, Universidad Politécnica de Madrid, 28040 Madrid, Spain; j.coteron@upm.es; 2 Departamento de Educación, Métodos de Investigación y Evaluación, Facultad de Ciencias Sociales y Humanas, Universidad Pontificia Comillas, 28049 Madrid, Spain; mcocete@comillas.edu; 3 Cátedra “Fundación Sanitas” de Estudios sobre Deporte Inclusivo, Facultad de Ciencias de la Actividad Física y del Deporte—INEF, Universidad Politécnica de Madrid, 28040 Madrid, Spain; j.perez@upm.es

**Keywords:** teachers’ antecedents, behavioural engagement, self-determination theory, autonomy support, multi-level analysis

## Abstract

Students’ engagement in Physical Education has been linked to several adaptive consequences. Even though the existing literature suggests that perceived autonomy support can predict engagement, research is scarce on how teachers’ antecedents might influence this behavioural outcome. This study sought to compare the influence of teachers’ basic psychological needs’ satisfaction and basic psychological needs’ thwarting on students’ behavioural engagement and on the relationship between perceived autonomy-support and the students’ behavioural engagement. The sample included 29 Physical Education teachers and 644 students who were taught by the participants teachers. Data were collected using both paper and online surveys and they were analysed using multilevel modelling techniques. The results revealed that, while teachers’ autonomy satisfaction might be significant in the explanation of students’ engagement (β = 0.33, *p* < 0.01), it seems that needs thwarting could be a better predictor of this outcome (β autonomy thwarting = −0.17, *p* < 0.01; β competence thwarting = −0.06, *p* < 0.05). These findings suggest the impact of certain external pressures on teachers’ practices which, in turn, might affect students’ behavioural outcomes.

## 1. Introduction

Engaging students in class activities is an important goal for teachers in physical education (PE) lessons. Student engagement is a multifaceted concept, reflecting behavioural, emotional, and cognitive aspects and it has been linked to diverse positive consequences [1,2]. Behavioural engagement, encompassing an array of behaviours including effort, exertion, and persistence as well as mental efforts such as concentration, attention, asking questions, and contributing to classroom discussions; has emerged as an important construct in the prediction of students’ performance and learning achievement and completion of school [3].

The existing research examines the social and intrapersonal processes associated with pupil behaviour, and self-determination theory (SDT) [4] has gained extensive empirical support within the domain of PE. The most relevant application of the SDT in PE is exhibited by the fact that the teacher can motivate students to engage and learn during class while providing needs support. In particular, it is posited that students will engage better and function more effectively in classroom environments that are perceived as autonomy-supportive [5,6]. Autonomy support refers to actively soliciting and nurturing children’s interests, providing choices and asking for input, allowing the expression of deviant points of view, making use of inviting language, providing meaningful rationales for requests, and respecting children’s pace of progress [7,8]. Whereas considerable research has demonstrated the advantages of teachers’ autonomy support, this literature has typically considered the students as the central group of interest [9]. Research that addresses teacher-related factors as reasons why teachers engage in this interpersonal style has been less forthcoming. A literature review carried out by Matosic, Ntoumanis and Quested in 2016 [10] the antecedents of need supportive and controlling interpersonal styles from a self-determination theory identify three broad categories of antecedents, namely contextual factors, perceptions of others’ behaviours and motivation, and personal factors. In line with this, some recent studies suggest that motivational variables for teachers could be relevant in the explanation of the interpersonal style they use when teaching [11,12].

Basic needs fulfilment has been proven to be a determinant of teachers’ autonomy-supportive behaviours, such as trying to gain an understanding of their students or providing them with instrumental help and support [13]. More recently, Korthagen and Evelein [14] found, in a study carried out with student teachers, that basic needs fulfilment was associated with more general adaptive teachers’ behaviours. Some authors have claimed that it is necessary to consider basic needs thwarting in regard to analysing maladaptive outcomes [15], and there is empirical evidence supporting the differentiation between a low need satisfaction and a need obstruction or frustration in a physical activity context [16]. However, to our knowledge, no previous studies have attempted to compare the influence of both needs satisfaction and needs thwarting on adaptive outcomes. This study fills a gap in the literature by examining how teachers’ need satisfaction and thwarting affect their teaching behaviours and students’ behavioural engagement.

### 1.1. Engagement in PE

Student engagement is a multifaceted concept [1]. In most studies, engagement is made up of two or three components although some studies include a fourth component when describing student engagement [17]. One of these components, behavioural engagement, has especially relevant implications for PE as it reflects distinct competences (such as effort, concentration or attention) which can promote long-term participation in physical activities [18].

Behavioural engagement has been defined as interactions with a setting that are active, goal driven, constructive and persistent [19,20]. When students interact with the setting in a positive way, they show several adaptive behaviours, such as putting great effort into their tasks, participating or following the teacher’s instructions [21,22].

The importance of student behavioural engagement in the academic context is reflected in its positive relationship with students’ grades and achievement [21] and in the risk of dropping out of the subject [23,24]. Dropping out of PE could be extended to limited physical activity due to the characteristics of the subject.

Given “the logical relationship of engagement to achievement and to optimal human development” [25], it is important to advance the research about this construct.

### 1.2. Teachers’ Provision of Autonomy Support in PE

Various studies have investigated student behavioural engagement as a positive consequence of the teachers’ way of interacting with students [26,27,28]. In every class, when teachers ask their students to engage in various learning activities, they unavoidably rely on a particular motivational style. Autonomy support is the most widely studied style [12,29,30]. Grounded in the SDT tenets, autonomy support is an interpersonal tone of support and understanding in which the teachers are empathetic and patient, foster students’ need satisfaction, provide rationales for their requests, use informational language and acknowledge and accept expressions of negative affect [31].

Over the last few years, several studies have focused on the understanding of PE teachers’ wellbeing and mental health [32,33] and some of these have demonstrated the existence of factors which explain why PE teachers orient themselves towards one motivational style rather than another. Some of these factors are the beliefs that teachers hold [8,34], the pressures they feel at their job [35,36], the perceived motivation in the students they teach [13,27] or their own personality disposition [37].

In more recent studies, researchers have become increasingly aware that teacher motivational variables also play an important role in the explanation of their motivational style [11,12].

### 1.3. Teachers’ Basic Psychological Needs Satisfaction and Thwarting

From a motivational perspective, and according to SDT, the fulfilment of the basic psychological needs (BPN) for autonomy, competence, and relatedness is critical, as these basic needs are said to serve as the psychological nutriments that energise personal growth and integrity [38]. Autonomy satisfaction refers to the need to express the authentic self and to experience the self as the source of action. When experiencing autonomy satisfaction in teaching, teachers might have room for their own ideas and choices and can develop them accordingly. Competence is based on the idea that organisms are born with the desire to experience mastery in what they do and, thus, influence their environment in a suitable way. This psychological nutriment could be recognised in teachers as the wish to feel able to manage their classrooms. Lastly, relatedness refers to the desire to experience care for other people and the feeling of belonging to a group [39].

There is strong evidence supporting that a teacher’s basic need satisfaction is not only relevant for their psychological functioning but also relates to the quality of their interactions with the students in PE class. In this line of inquiry, Taylor et al. [13] suggested that the BPN satisfaction experienced by teachers could predict some motivational strategies, such as providing support or gaining an understanding of students both directly and indirectly through self-determined motivation. Carson and Chase [40] found a connection between BPN satisfaction in teachers and some professionalism behaviours, such as conference attendance or reading journals. Considering the current advances in this issue, it can be hypothesised that teachers are more likely to experience adaptive interactions with their students. In a more recent study, Abós et al. [11] suggest the influence of BPN satisfaction on some autonomy-supportive behaviours, such as explaining the relevance of a task, involving students in decision-making or showing interest in students’ preferences.

Although BPN satisfaction has been more thoroughly studied, Ryan and Deci [41] proposed that the thwarting of BPN will lead to non-optimal development and ill health. Need thwarting does not simply reflect the perception that need satisfaction is low but is a feeling that psychological needs are being obstructed or actively frustrated. Despite having been widely discussed in theoretical overviews, little research has focused on the direct consequences of psychological need thwarting. In the athletic domain, it has been found that athletes’ need satisfaction can be predicted by autonomy-supportive behaviours whilst need thwarting can better be predicted by coach control [42]. The scarce works that have addressed the study of this construct among PE teachers have tested the association between BPN thwarting and some maladaptive outcomes, such as amotivation, burnout or somatic complaints [15,43].

To the best of our knowledge, no previous studies have aimed to explain how BPN thwarting among teachers might be related to behavioural outcomes affecting the learning process. Considering the differentiation between need thwarting and the lack of need satisfaction among teachers, understanding the role of both constructs in the prediction of positive motivational consequences could add valuable knowledge to the existing literature. Therefore, the main purpose of this study is to compare the association between teachers’ BPN satisfaction with students’ behavioural engagement and teachers’ BPN thwarting with students’ behavioural engagement; as well as the relationship of these constructs with the association between perceived autonomy-support and the students’ behavioural engagement.

## 2. Method

### 2.1. Participants and Procedure

The sample was composed of 29 PE teachers (65.5% men, *M*_age_ = 45.38; *SD*_age_ = 9.00) from 27 private high schools in the city of Armenia, Colombia. These teachers had on average approximately 18 (*M* = 17.93, *SD* = 9.78) years of experience. One group of students among those taught by each of the participant teachers was randomly selected; thus, a total of 644 students aged between 12 and 16 (46.9% boys, *M*_age_ = 15.16; *SD*_age_ = 1.78) took part in the study. Students attended basic secondary school (equivalent to middle school) and belonged to families of a medium- and low-socioeconomic level.

After receiving approval from the University Ethics Committee, all participants were treated in agreement with the ethical guidelines of the American Psychological Association [44] with respect to consent, confidentiality and anonymity of their answers. Both teachers and students were informed about the study and consented to participate. Moreover, informed written consent was obtained from the parents and the head teachers of the schools on behalf of the children involved in the study. Teachers were asked to answer an online questionnaire so that they could take their time to reflect about their answers. At the beginning of a scheduled PE lesson, students were asked to honestly answer the questions in the study questionnaire and were told that there were no right or wrong answers. The participants required approximately 20 min to complete the questionnaire, which was administered by research assistants in quiet classroom settings without the PE teachers present.

### 2.2. Measures

#### 2.2.1. Teachers’ Basic Needs Satisfaction

Teachers’ perceptions of need satisfaction were assessed using an adapted and translated version of the 21-item Basic Need Satisfaction at Work Scale [45]. The stem used in the questionnaire was “Rate the following statements”, and teachers rated the extent to which they felt their psychological needs for autonomy (e.g., “I feel like I can pretty much be myself at work”), competence (e.g., “Most days I feel a sense of accomplishment from working”), and relatedness (e.g., “People at work care about me”) were satisfied in the teaching context. Each of the three subscales were composed of 7 items, and the responses were provided on a 5-point scale ranging from 1 (strongly disagree) to 5 (strongly agree). The results of the confirmatory factor analysis carried out with the present study sample yielded adequate model goodness of fit indices (χ^2^_24_ = 56.88, *p* < 0.001, χ^2^/df = 2.37, CFI = 0.91, IFI = 0.92, SRMR = 0.06). The internal consistency coefficient alpha values were all greater than 0.73. The scale is provided in the Table S1.

#### 2.2.2. Teachers’ Basic Needs Thwarting

Teachers’ perceptions of need thwarting were assessed using the Spanish version adapted for teachers [46] of the 12-item Psychological Need Thwarting Scale (PNTS) [47]. The stem used in the questionnaire was “In my PE classes…”, and teachers rated the extent to which they felt their psychological needs for autonomy (e.g., “I feel prevented from making choices with regard to the way I teach”), competence (e.g., “Situations occur in which I am made to feel inadequate”), and relatedness (e.g., “I feel other people dislike me”) were thwarted in the teaching context. Each of the three subscales were composed of 4 items, and the responses were provided on a 7-point scale ranging from 1 (strongly disagree) to 7 (strongly agree). The results of the confirmatory factor analysis carried out by Cuevas et al. [46] yielded adequate model goodness of fit indices (χ^2^_51_ = 248.61, *p* < 0.001, χ^2^/df = 4.87, CFI = 0.95, IFI = 0.96, SRMR = 0.05). In this same study the internal consistency coefficient alpha values were all greater than 0.81. The scale is provided in the Table S2. 

#### 2.2.3. Students’ Behavioural Engagement

Students’ behavioural engagement was assessed using an adapted and translated version of the dimension of behavioural engagement included in the instrument designed by Shen et al. [48] to measure students’ perceptions of their effort, attention, and persistence in PE classes. The stem used in the questionnaire was “When I am in PE classes…”, and it was followed by five items grouped in a single dimension (e.g., “I listen to the teacher very carefully”). The responses were provided on a 5-point scale ranging from 1 (strongly disagree) to 5 (strongly agree). The results of the confirmatory factor analysis carried out with this study sample yielded adequate model goodness of fit indices (χ^2^_5_ = 51.03, *p* < 0.001, χ^2^/df = 10.21, CFI = 0.91, IFI = 0.91, SRMR = 0.06). A high Cronbach alpha value provided further validation support for the internal consistency of this scale (α = 0.83). The scale is provided in the Table S3.

#### 2.2.4. Students’ Perceived Autonomy Support

Students’ perception of autonomy support was assessed using the Spanish version [49] of the scale designed by Röder and Kleine [50]. The stem used in the questionnaire was “When I am in PE classes…”, and it was followed by five items grouped in a single dimension (e.g., “We can often choose between different tasks”). The responses were provided on a 5-point scale ranging from 1 (strongly disagree) to 5 (strongly agree). The results of the confirmatory factor analysis carried out by Ruiz (2015) yielded adequate model goodness of fit indices (χ^2^_10_ = 11.79, *p* < 0.05, χ^2^/df = 2.35, CFI = 0.99, IFI = 0.99, RMSEA = 0.05). In this same study the internal consistency coefficient alpha for this scale was 0.87. The scale is provided in the Table S4.

### 2.3. Data Analysis

**Figure 1:**
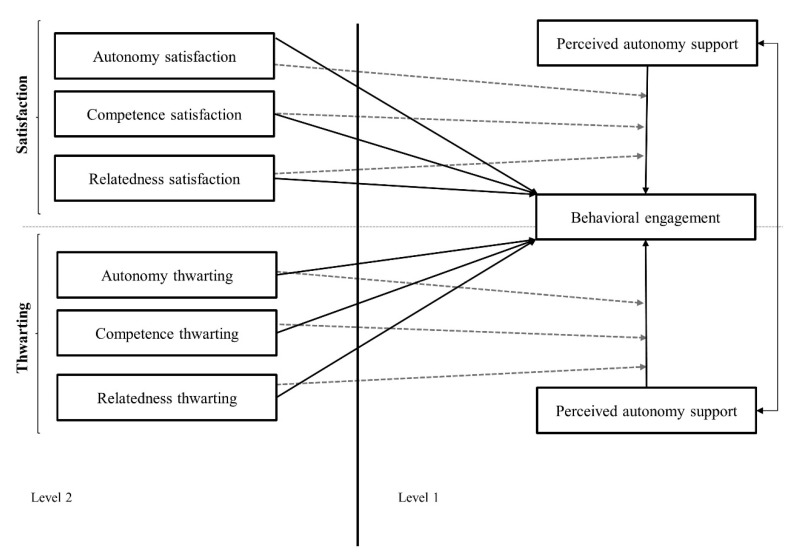
Multilevel models tested.

Prior research investigating engagement has mainly used methods and analyses that assume that students’ perceptions are independent of one another or have no influence on other students in the same group. However, students in the same group share experiences with each other and with teachers that make them more similar, thus violating the assumption of independence. In the case of nested data, multilevel modelling (MLM) is a statistical technique that takes into account the contextual variable of group membership and alleviates issues related to violating the assumption of independence. For the present study, students were nested within groups which were taught by the same PE teacher. Following a previously used procedure [51], two multilevel analyses were therefore performed to examine the relationship between (a) the teachers’ BPN satisfaction and students’ perceived autonomy support with students’ behavioural engagement, and (b) the teachers’ BPN thwarting and students’ perceived autonomy support with students’ behavioural engagement (see Figure 1). For each of the analyses, three models were computed in which independent variables were cumulatively aggregated in the models. Integrating independent variables, step by step, allowed us to investigate how the inclusion of the variables influences the effects of previously included independent variables on the outcome variable.

First, a null model (equivalent to a one-way ANOVA with random effects) was tested. Second, a random intercepts model was tested by adding the level-1 predictor, which was students’ perceived autonomy support (Model 1). Finally, the level-2 predictors were included in the model (Model 2). In the first multilevel analysis, predictors were basic needs’ satisfaction, while in the second multilevel analysis, basic needs’ thwarting were used as predictors.

The level-1 predictor was group-mean centred, and the level-2 predictors were grand-mean centred to interpret the potential cross-level interaction [52]. For all variables in the final model, an effect size based on the formula that Elliot and Simmons recommend for multilevel models was reported [53]. Changes in deviances (calculating −2 LL differences via Chi-square tests) were examined across different models to decide the best-fitting model and whether the fixed effect and/or random effect should be added.

Data entry, screening and cleaning, assumptions testing, descriptive statistics, and calculation of reliability coefficients were conducted using SPSS 24 (IBM Corporation, Armonk, NY, USA). Multi-level models were created in HLM 7 (Scientific Software International, Inc., Skokie, IL, USA).

## 3. Results

### 3.1. Descriptive Statistics

Descriptive statistics and intercorrelations for all study variables are presented in Table 1. The correlation coefficients were in the expected direction and ranged in effect size from small to medium. Thus, satisfaction of all BPN positively correlated with each other, and there were also positive correlations between thwarting of the three BPN. Students’ engagement correlated positively with BPN satisfaction and negatively with BPN thwarting. As shown in Table 1, associations between students’ engagement and the BPN thwarting were stronger than those between students’ engagement and BPN satisfaction were. Finally, autonomy support perceived by students was positively associated with autonomy satisfaction and negatively correlated with autonomy and competence thwarting.

### 3.2. Prediction of Students’ Engagement through BPN Satisfaction

#### 3.2.1. Null Model

First, an ANOVA was performed to confirm that the variability in the outcome variable, by the level 2 group, was significantly different than zero. A chi-square test revealed the existence of variance in engagement by the level-2 groupings (χ^2^_28_ = 289.58, *p* < 0.001). This result indicates that there is statistical justification for running HLM analyses. This model showed an intraclass correlation coefficient (ICC) of 0.31. Thus, this value indicates that 31% of the variance in behavioural engagement is between-groups, and 69% of the variance in engagement is between students within a given group. The existence of variance at both levels justified the introduction of predictors at each level.

#### 3.2.2. Random Intercepts Model

Next, the relationship between the level 1 predictor variable (perceived autonomy support) and the outcome variable (behavioural engagement) was tested. In this model, each group of an individual class is the subject variable under which students are grouped. This model explores whether the level-2 effect discovered in the null model may be attributed in part to some groups of students perceiving higher autonomy support than others. As shown in Table 2, the regression coefficient relating perceived autonomy support to behavioural engagement was positive and statistically significant (B = 0.08, *p* < 0.01). Thus, the higher the autonomy support perceived by students was, the more behavioural engagement they showed. The proportion variance explained at level-1 was 3%. Thus, 3% of the level-1 variance in outcome is accounted for by the perception of autonomy support by students.

#### 3.2.3. Intercepts- and Slopes-as-Outcomes Model

Finally, the intercepts model and slopes-as-outcomes model were simultaneously tested with all predictor variables in the model to check for the presence of any interactions between predictor variables. The regression coefficient relating teachers’ autonomy satisfaction to students’ behavioural engagement was positive and statistically significant (B = 0.33, *p* < 0.01). However, the regression coefficients were not significant in the case of teachers’ competence satisfaction (B = 0.12, *p* > 0.01) or teachers’ relatedness satisfaction (B = −0.01, *p* > 0.01). The cross-level interactions between teacher’s need satisfaction and perceived autonomy support were not statistically significant (B_Autonomy_ = 0.02, *p* < 0.05; B_Competence_ = −0.05, *p* < 0.05; B_Relatedness_ = 0.02, *p* < 0.05), which means that the degree of the teachers’ needs satisfaction was not related to the strength of the relationship between perceived autonomy support and behavioural engagement. Regression coefficients and standard errors of this model are shown in Table 2.

### 3.3. Prediction of Students’ Engagement through BPN Thwarting

Next, the model in which students’ behavioural engagement is predicted by their perception of autonomy as well as by their teachers’ needs thwarting was tested. The same steps previously described for the model including needs satisfaction, were followed. Thus, the first two models (unconstrained and random intercepts models) were identical to those described in the previous section. New information is provided, however, for the intercepts- and slopes-as-outcomes model.

#### Intercepts- and Slopes-as-Outcomes Model

Finally, the intercepts model and slopes-as-outcomes model were simultaneously tested with all predictor variables in the model to test the presence of any interactions between predictor variables. The regression coefficient relating teachers’ autonomy thwarting (B = −0.17, *p* < 0.001) and teachers’ competence thwarting (B = −0.06, *p* < 0.05) to students’ behavioural engagement were both negative and statistically significant. However, the regression coefficient was not significant in the case of the teachers’ relatedness thwarting (B = −0.03, *p* > 0.01). The cross-level interactions between teachers’ need thwarting and perceived autonomy support were not statistically significant (B_Autonomy_ = 0.02, *p* < 0.05; B_Competence_ = −0.04, *p* < 0.05; B_Relatedness_ = 0.02, *p* < 0.05), which means that the degree of teachers’ needs thwarting was not related to the strength of the relationship between perceived autonomy support and behavioural engagement. Regression coefficients and standard errors are shown in Table 3.

## 4. Discussion

The main purpose of this study was to compare the relationship between teachers’ BPN satisfaction and BPN thwarting with students’ behavioural engagement as well as the relationship of these constructs with the association between perceived autonomy-support and the students’ behavioural engagement. The value of the study was provided by analysing constructs associated with both students and teachers, which allowed us to examine the top-down association between teacher-level constructs (BPN satisfaction and thwarting) and the individual-level constructs of perceived autonomy support (PAS) and behavioural engagement. Furthermore, the study aimed to gain knowledge on the different effects that BPN satisfaction and thwarting have on students’ relevant outcomes.

With respect to the students’ level correlates, the findings of the present study confirmed an effect of PAS on behavioural engagement. Behavioural engagement reflects the extent to which students listen to the teacher, enjoy the activities proposed, show effort and persistence or answer the teacher’s questions. As previous studies have shown, when students report a high perception of autonomy support from teachers, they view their class activities as volitional and self-determined and engage in PE class willing and eagerly [6,54,55]. It has been suggested that this relationship could be mediated by autonomous motivation and/or positive emotions [6,56]. In any case, this finding does have practical implications for teaching PE. Thus, to enhance autonomy-supportive environments, teachers should carry out certain instructional behaviours, such as listening to the students’ perspective, vitalising inner motivational resources, using informational language, providing explanatory rationales, acknowledging and accepting negative affect and displaying patience [31,57].

While it is widely accepted that when teachers display greater autonomy support their students show relevant and wide-ranging gains in adaptive outcomes [31,58], the literature has not yet been able to explain precisely what and how antecedents in teachers might affect both the environment and students’ outcomes.

The findings of the present study showed that motivational variables among teachers might play an important role in the explanation of behavioural outcomes in students. More specifically, both BPN satisfaction and thwarting in teachers were tested as predictors of students’ engagement.

On the one hand, our results revealed that teachers’ autonomy satisfaction would affect students’ behavioural engagement, but competence and relatedness satisfaction would have no effect on this type of engagement. Aelterman, Vansteenkiste, Van Keer and Haerens [59] carried out a study aiming to examine whether PE teachers’ psychological need satisfaction experienced during continuous professional development (CPD) on need-supportive teaching predicted changes in their effectiveness and feasibility beliefs regarding the proposed teaching approach, and their intentions to apply this approach and the subsequent changes in their self-reported in-class behaviours were also evaluated. Interestingly, it was found that experiencing need satisfaction did not only relate to a change in teachers’ beliefs but was also directly associated with teachers’ intentions to apply the proposed teaching strategies. More recently, Cheon, Reeve, Lee, et al. [12] investigated what resources teachers acquired during an autonomy-supportive intervention programme that explained their ability to successfully upgrade the quality of their motivational style. The findings from this study did not support greater need satisfaction as a mediating process. Neither Aelterman et al. or Cheon et al. examined independent associations between each of the three basic needs and teaching outcomes. It could be inferred from the findings of the present study that particularly the experience of autonomy satisfaction by PE teachers plays a determinant role in the explanation of behavioural engagement in students. While Aelterman et al. assessed BPN satisfaction during CPD, our study focused on the analysis of BPN during teaching practice. The findings of the present study suggest that the satisfaction of autonomy, when considered as an independent variable, seems to have positive effects on teaching practice regardless of its source. In addition, our study proves the direct association between autonomy satisfaction experienced by teachers and students’ behavioural engagement.

Thus, it seems to be a practical implication that fostering teachers’ autonomy during their daily activities could lead to greater student engagement during classes. For instance, providing teachers with room to make their own decisions and address their feelings and needs might be key elements to fostering the development of a PAS environment and, thus, to students’ behavioural engagement.

On the other hand, apart from analysing the effect of teachers’ BPN satisfaction on students’ outcomes, the present study aimed to compare BPN satisfaction and thwarting regarding their potential to predict behavioural engagement in students.

There is some evidence in the existing literature that has addressed the analysis of BPN thwarting among teachers [15,43,46]. The findings from these investigations revealed that BPN thwarting could mediate the relationship between pressures perceived by PE teachers and burnout in this population. However, the effect that BPN thwarting in teachers could have on learning experiences has not been examined.

Despite the existence of evidence suggesting that need satisfaction and need thwarting may be part of a continuum [60], more recent research has concluded that they are two distinguishable constructs that should be addressed independently, as they exhibit different relationships to external outcomes [61,62,63]. As Bartholomew et al. suggested, need thwarting is characterised by a perception that the psychological needs are obstructed or actively undermined [64].

In the educational context, a teacher might not feel autonomous because they perceive that their opinions are not considered in their work setting or because they feel pressure to behave and teach in a certain way. The first situation is a case of low need satisfaction, as there is no sign of active psychological needs obstruction, whereas the latter is a case of need thwarting, as it implies that the teacher’s autonomy is being actively undermined. In the case of competence, teachers may not feel competent because they have scarce knowledge about the content that they are teaching or because they do not have a place to display their skills. Again, the first situation is a case of low need satisfaction, as there is no sign of active psychological needs obstruction, whereas the latter is a case of need thwarting, as it implies that teacher’s competence is being actively undermined.

The findings of the present study are in line with this second approach, which does not consider satisfaction and thwarting as a part of a continuum since associations between BPN satisfaction and thwarting with behavioural engagement were different. With respect to BPN thwarting, our study sheds light on the issue suggesting that both autonomy and competence thwarting in teachers could influence students’ behavioural engagement.

According to our results, while both autonomy satisfaction and thwarting seem to have a significant effect on students’ behavioural engagement, in the case of competence it is the thwarting that might better explain students’ engagement. Teachers could feel pressure from different sources that make them feel that they are not allowed to show their own competence. For instance, time constraints could make teachers believe that they do not have the ideal conditions to develop learning situations, even though they might feel skilled enough to do so. Furthermore, the increasing of administrative tasks might undermine teachers’ competence because they feel forced to perform duties they have not been trained for. In addition, restrictive norms with respect to the use of teaching methodologies might be likely to foster teachers’ competence thwarting. A teacher could have a vast knowledge about the problem-based approach to teach PE, but their perception of competence might be undermined if the authorities at the school push their teachers to follow a certain cooperative learning approach.

Finally, neither relatedness satisfaction nor thwarting in teachers significantly explained the students’ engagement. Despite being one of the three BPN, relatedness has often played a less determinant role than autonomy and competence needs in studies developed among teachers through the lens of the trans-contextual model. Sánchez-Oliva et al. [43] suggested that relatedness thwarting in PE teachers was not a predictor of amotivation. Other studies have found relatedness thwarting to be the least important BPN in the explanation of teachers’ burnout [46] and somatic complaints [15].

Teachers’ relatedness has usually been defined as connections to colleagues. This understanding of the construct is reflected in the items that composed the questionnaire which was used to measure this variable in the study (e.g., “I feel other people dislike me”). Since it has been proven that satisfaction of the need for relatedness with students leads to higher levels of engagement and positive emotions than does satisfaction of the need for relatedness with peers [65], it would be interesting to further analyse the role of a reconceptualised relatedness dimension taking into account connections between teachers and students. Since research investigating teachers’ motivation to enter teaching has shown that the opportunity to work closely with students is a strong motive for entering the profession [66], this new approach could give relatedness a more relevant role in the explanation of subsequent teachers’ emotions and behaviours.

It would be interesting for future research to explore the factors that can affect teachers’ perceptions of both need satisfaction and thwarting and to investigate whether school-level interventions can be effective in the improvement of teachers’ motivation. On the other hand, it would be helpful to gain some more fine-grained understanding of the consequences that teachers’ motivation might have on their practice. In this respect, future studies could examine the associations between teachers’ motivation and teachers’ interpersonal styles using the recent advances pointed out by the circumplex approach [67].

We note three limitations to the present research. First, the sample of teachers was confined to a single country. Therefore, the generalisability of the current findings to other countries is unknown and should be explored. Second, even though both teachers’ and students’ data were collected, the study was cross-sectional in nature, which prevented us from testing causal relationships between variables. Consequently, new experimental studies could complement the results of our study by testing the effect of actively fostering teachers’ BPN on their motivational style and, in turn, students’ behavioural outcomes. Third, the operationalisation of relatedness satisfaction and thwarting as connections with colleagues only could have led to misinformation regarding how the relationships with students as perceived by teachers might affect their teaching practice and, in turn, students’ outcomes. It would be interesting to further explore relatedness from a teacher-student perspective.

## 5. Conclusions 

To conclude, our study findings showed that while teachers’ autonomy satisfaction might be significant in the explanation of students’ behavioural outcomes (engagement), it seems that needs thwarting could be a better predictor of these outcomes. Specifically, while teachers’ competence satisfaction was not found to be associated with students’ behavioural engagement, teachers’ competence thwarting did emerge as a predictor of this construct. This finding has some interesting implications. Firstly, that certain pressures affecting teachers’ perceptions of autonomy might indirectly lead to maladaptive interactions with students which, in turn, can affect students’ engagement. The root of autonomy thwarting feelings could be, for instance, in the existence of a too close and controlling prescriptive curriculum under which the teacher is forced to comply with. Another possible trigger of the perception of a lack of autonomy, could rely on the nature of the subject. PE teachers are frequently working in locations which are visible to other teachers and authorities at school and this might explain why PE teachers feel particularly stressed to conform to certain methodologies. On the other hand, it is worth mentioning the fact that PE is often undervalued [68]. This negative attitude can cause school management and colleagues to engage in behaviours, either deliberately or not, that actively undermine teachers’ psychological needs of competence. Considering this, it seems that making school principals aware of the importance of being attentive to the work carried out by PE teachers and provide them with recognition and support is essential to improve teachers’ well-being and, in turn, the quality of their teaching practices.

## Figures and Tables

**Table 1:** Descriptive statistics, between-level and within-level correlations between study variables.

Variable	1	2	3	4	5	6	7	8
1. Autonomy satisfaction								
2. Competence satisfaction	0.45 **							
3. Relatedness satisfaction	0.49 **	0.46 **						
4. Autonomy thwarting	−0.53 **	−0.22 **	−0.24 **					
5. Competence thwarting	−0.43 **	−0.51 **	−0.28 **	0.61 **				
6. Relatedness thwarting	−0.36 **	−0.55 **	−0.39 **	0.43 **	0.56 **			
7. Engagement	0.29 **	0.22 **	0.17 **	−0.48 **	−0.40 **	−0.31 **		
8. Students’ autonomy support	0.15 **	0.04	−0.01	−0.28 **	−0.16 **	−0.07	0.27 **	
Possible range	1–5	1–5	1–5	1–7	1–7	1–7	1–5	1–5
*M*	4.01	4.11	3.76	2.36	2.03	1.87	4.21	3.01
*SD*	0.53	0.61	0.48	1.47	1.34	1.10	0.65	1.04

Note: ** = *p* < 0.01.

**Table 2:** Two-level multilevel analysis with behavioural engagement as a dependent variable and teacher’ need satisfaction as level-2 predictors.

Parameter		Null Model	Model 1	Model 2	Effect Size
**Fixed part**					
	Intercept	4.19 (0.07)	4.19 (0.07)	4.19 (0.06)	
	**Student level variables (level1)**				
	PAS		0.08 (0.03) **	0.08 (0.02) **	0.017
	**Teacher level variables (level2)**				
	Autonomy satisfaction			0.33 (0.14) *	0.318
	Competence satisfaction			0.12 (0.12)	
	Relatedness satisfaction			−0.01 (0.16)	
	**Interaction**				
	Autonomy satisfaction * slope			0.02 (0.05)	
	Competence satisfaction * slope			−0.05 (0.05)	
	Relatedness satisfaction * slope			0.02 (0.07)	
**Random part**					
	**Teacher-level variance**	0.37 (0.14)	0.37 (0.14)	0.32 (0.10)	
	**Student-level variance**	0.55 (0.30)	0.54 (0.29)	0.54 (0.29)	
**Model fit**					
	**Deviance**	1117.54	1114.83	1093.42	
	**χ^2^/df**		2.71 (2)	21.41 (8) **	

Note: *n* (students) = 644 and *n* (teachers) = 29; Effect size = Effect size for model 2; Per cell: regression coefficient (standard errors); * = *p* < 0.05; ** = *p* < 0.05. PAS = perceived autonomy support.

**Table 3:** Two-level multilevel analysis with behavioural engagement as a dependent variable and teachers’ need thwarting as a level-2 predictor.

Parameter		Null Model	Model 1	Model 2	Effect Size
**Fixed part**					
	Intercept	4.19 (0.07)	4.19 (0.07)	4.19 (0.03)	
	**Student level variables (level1)**				
	PAS		0.08 (0.03) **	0.10 (0.02) **	0.014
	**Teacher level variables (level2)**				
	Autonomy thwarting			−0.17 (0.03) ***	0.036
	Competence thwarting			−0.06 (0.03) *	0.012
	Relatedness thwarting			−0.03 (0.03)	
	**Interaction**				
	Autonomy thwarting * slope			−0.02 (0.02)	
	Competence thwarting * slope			0.04 (0.03)	
	Relatedness thwarting * slope			0.02 (0.03)	
**Random part**					
	**Teacher-level variance**	0.37 (0.14)	0.37 (0.14)	0.11 (0.02)	
	**Student-level variance**	0.55 (0.30)	0.54 (0.29)	0.54 (0.29)	
**Model fit**					
	**Deviance**	1117.54	1114.83	1053.71	
	**χ^2^/df**		2.71 (2)	61.12 (8) ***	

Note: *n* (students) = 644 and *n* (teachers) = 29; Effect size = Effect size for model 3; Per cell: regression coefficient (standard errors); * = *p* < 0.05; ** = *p* < 0.01; *** = *p* < 0.001. PAS = perceived autonomy support.

## Supplementary Materials

The following are available online at https://www.mdpi.com/1660-4601/17/22/8573/s1, Table S1: Teachers’ need satisfaction, Table S2: Teachers’ need thwarting, Table S3: Students’ behavioural engagement, Table S4: Students’ perceptions of autonomy support.
