# Supplementary material for: Teachers’ Psychological Needs Satisfaction and Thwarting: Can They Explain Students’ Behavioural Engagement in Physical Education? A Multi-Level Analysis
Source: Int J Environ Res Public Health. 2020 Nov 19;17(22):8573. doi: 10.3390/ijerph17228573 (PMC7699264; doi:10.3390/ijerph17228573)
Supplement: Supplementary file 1 [file ijerph-17-08573-s001.pdf]

Supplementary Materials. Instruments used in the study

Table S1 Teachers' need satisfaction.

Valora las siguientes afirmaciones:

1	Siento que tengo un amplio margen para decidir cómo hacer mi trabajo	1	2	3	4	5
2	Realmente me gusta la gente con la que trabajo	1	2	3	4	5
3	No me siento muy competente cuando estoy en el trabajo	1	2	3	4	5
4	La gente en el trabajo me dice que soy bueno/a en lo que hago	1	2	3	4	5
5	Me siento presionado/a en el trabajo	1	2	3	4	5
6	Me llevo bien con la gente del trabajo	1	2	3	4	5
7	Soy muy reservado/a cuando estoy en el trabajo	1	2	3	4	5
8	Soy libre para expresar mis ideas y opiniones en el trabajo	1	2	3	4	5
9	Considero que la gente con la que trabajo son mis amigos	1	2	3	4	5
10	He sido capaz de aprender nuevas e interesantes habilidades en mi trabajo	1	2	3	4	5
11	Cuando estoy en el trabajo tengo que hacer lo que me dicen	1	2	3	4	5
12	La mayor parte del tiempo tengo una sensación de logro en mi trabajo	1	2	3	4	5
13	Mis sentimientos son tenidos en cuenta en el trabajo	1	2	3	4	5
14	En mi trabajo no tengo muchas oportunidades de demostrar lo capaz que soy	1	2	3	4	5
15	La gente en el trabajo se preocupa por mí	1	2	3	4	5
16	No soy muy cercano/a a mucha gente en mi trabajo	1	2	3	4	5
17	Siento que puedo ser yo mismo/a en el trabajo	1	2	3	4	5
18	No me agrada mucho la gente con la que trabajo	1	2	3	4	5
19	Cuando estoy trabajando, con frecuencia no me siento muy capaz	1	2	3	4	5
20	No hay muchas oportunidades para decidir por mí mismo/a cómo hacer mi trabajo	1	2	3	4	5
21	La gente en el trabajo es muy amable conmigo	1	2	3	4	5

Autonomy satisfaction: 1, 5, 8, 11, 13, 17, 20

Competence satisfaction: 3, 4, 10, 12, 14, 19

Relatedness satisfaction: 2, 6, 7, 9, 15, 16, 18, 21

Table S2 Teachers' need thwarting.

En mi ambiente de trabajo

1	Siento que me impiden tomar decisiones respecto al modo en el que enseño	1	2	3	4	5	6	7
2	Me siento presionado/a a comportarme de determinada manera	1	2	3	4	5	6	7
3	Me siento forzado/a a seguir una determinada forma de enseñar	1	2	3	4	5	6	7
4	Me siento presionado/a a aceptar las formas de enseñanza que me han estipulado	1	2	3	4	5	6	7
5	Hay situaciones que me hacen sentir incapaz	1	2	3	4	5	6	7
6	A veces digo cosas que me hacen sentir incompetente	1	2	3	4	5	6	7
7	Hay situaciones que me hacen sentir torpe	1	2	3	4	5	6	7
8	Siento que no estoy a la altura porque no tengo oportunidades para demostrar mi potencial	1	2	3	4	5	6	7
9	Siento que soy rechazado/a por aquellos que me rodean	1	2	3	4	5	6	7
10	Siento que los demás pueden ser indiferentes conmigo	1	2	3	4	5	6	7
11	Siento que la gente de mi centro educativo no me agrada	1	2	3	4	5	6	7
12	Siento que otros tienen envidia cuando logro éxitos	1	2	3	4	5	6	7

Autonomy thwarting: 1, 2, 3, 4

Competence thwarting: 5, 6, 7, 8

Relatedness thwarting: 9, 10, 11, 12

Table S3 Students' behavioural engagement

En clase de educación física…

1. Me esfuerzo para hacerlo bien	1	2	3	4	5
2. Trabajo tanto como puedo	1	2	3	4	5
3. Participo en las actividades	1	2	3	4	5
4. Presto atención	1	2	3	4	5
5. Escucho muy atentamente	1	2	3	4	5

Table S4 Students' perceptions of autonomy support

En clase de educación física…

1. A menudo, podemos decidir entre varios temas	1	2	3	4	5
2. A menudo, podemos decidir si queremos trabajar solos o en grupos	1	2	3	4	5
3. A menudo, podemos decidir la forma de trabajar los temas, por ejemplo con un libro, vídeo, grupos de discusión,…	1	2	3	4	5
4. A menudo, podemos decidir cuándo y cuánto tiempo trabajamos en una tarea	1	2	3	4	5
5. A menudo, podemos decidir entre diferentes tareas	1	2	3	4	5
